# Improving screening for disordered eating behaviors in pediatric weight management using quality improvement methodology

**DOI:** 10.1016/j.obpill.2025.100198

**Published:** 2025-07-22

**Authors:** Roohi Y. Kharofa, Sanita L. Ley, Kristin M. Stackpole, Jessica A. Lin, Carolina M. Bejarano, Katelyn Gordon, Shelley Kirk, Melissa Burbrink, Robert M. Siegel

**Affiliations:** a Center for Better Health and Nutrition, The Heart Institute, Cincinnati Children's Hospital Medical Center, 2800 Winslow Avenue, Cincinnati, OH, 45206, USA; b University of Cincinnati College of Medicine, 3230 Eden Ave, Cincinnati, OH, 45267, USA; c Division of Behavioral Medicine & Clinical Psychology, Cincinnati Children's Hospital Medical Center, 3333 Burnet Ave, Cincinnati, OH, 45229, USA; d Division of Adolescent and Young Adult Medicine, Cincinnati Children's Hospital Medical Center, 3333 Burnet Ave, Cincinnati, OH, 45229, USA

**Keywords:** Disordered eating behavior, Pediatric obesity, Screening, Quality improvement

## Abstract

**Background:**

Adolescents with obesity are at increased risk for disordered eating behaviors (DEBs). Despite screening being recommended, identification of DEBs in pediatric weight management (PWM) remains inadequate.

**Methods:**

The aim of this quality improvement project was to increase provider documentation of screening for DEBs from 44 % to 80 % for new patients, age ≥12, seen at a PWM program over a 10-month period. Interventions were grouped into four PDSA cycles: 1. Provider education, 2. Screening tool implementation, 3. Systematic use of screening tool, and 4. Implementation of an electronic screener. The primary outcome was the percent of patients with documentation of DEB screening. Data was plotted on a p-chart. Standard statistical process control methods were used.

**Results:**

Mean documentation rates for DEBs increased from a baseline of 44 %–85 %, with special cause analysis demonstrating improvement. Referrals for DEBs increased from 13 % at baseline to 19 %.

**Conclusion:**

Consistently screening for disordered eating behaviors in pediatric weight management is feasible. Quality improvement methodology can be utilized to help identify and act on facilitators and barriers to screening. Improved screening may lead to earlier identification of high-risk patients and prompt referral to mental health practitioners.

## Graphical abstract

**Figure:**
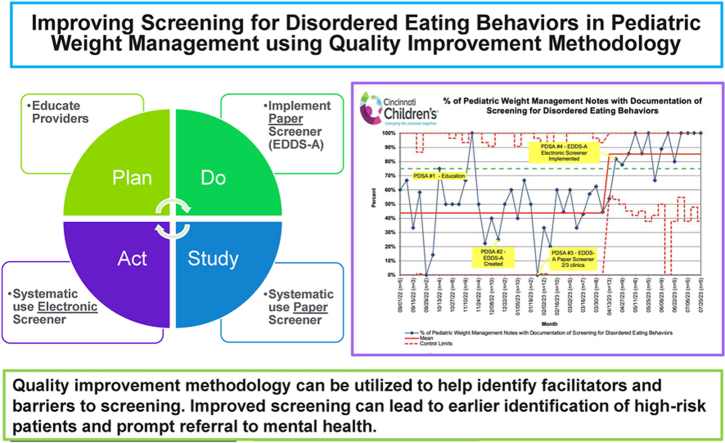

## 1 Introduction

A clear association exists between obesity and disordered eating behaviors (DEBs) [1,2]. Both the American Academy of Pediatrics [3] and the Obesity Medicine Association [4] recommend screening for DEBs in adolescents with obesity. Early Identification of DEBs (i.e., both subclinical disordered eating and clinically diagnosable eating disorders) is key to creating a comprehensive treatment plan focused on healthy eating and lifestyle patterns (not solely weight loss), and if needed, timely referral to an eating disorder specialist (physician and/or psychologist), which has been associated with improved prognosis for both DEBs and obesity [2,5,6]. Unfortunately, underdiagnosis and delayed diagnosis of DEBs in patients with obesity is common [7,8]. Our pediatric weight management (PWM) program has a dedicated psychologist, and we work closely with our institution's eating disorder program (EDP) to refer patients when DEBs are identified. Despite these resources, screening for DEBs within PWM was inconsistent at our site. To improve screening and patient outcomes, our PWM program initiated a quality improvement (QI) project aimed at increasing screening and documentation of DEBs for adolescents (≥12 years) seen for a new PWM visit from 44 % to 80 % over a 10-month period.

## 2 Methods

### 2.1 Context

This was a retrospective analysis based on a QI initiative. This work was undertaken at a single PWM program located within a large children's hospital. Patients are seen at the main hospital as well as 11 satellite locations. In fiscal year 2024, the clinic completed over 3000 medical visits, of which approximately 20 % were new visits. The clinic population is 51 % female, 47 % white, 38 % black, 18 % Hispanic and 48 % Medicaid-insured. The program includes 3 pediatricians board-certified in obesity medicine, 5 dietitians, 4 exercise physiologists and a child and adolescent psychologist with expertise in obesity and DEBs. The QI team consisted of the 3 obesity medicine pediatricians, the team psychologist, an additional psychologist, two adolescent medicine physicians from the EDP, a dietitian, a nurse, and a QI consultant. Prior to this QI initiative, some medical providers in the PWM program screened for DEBs verbally (no screening tool) and the number/type of questions varied.

To determine how to improve screening, the QI team created a process map [9] that revealed the following flow when DEBs were identified by a provider: 1) Documentation of DEBs in the history of the clinical note, 2) Documentation in the assessment/plan, 3) Verbal discussion with the dietitian, and 4) Referral to psychology and/or EDP. It was determined, based on expert consensus of the QI group, that the ideal process would always include Step 1. Steps 2 and 3 would be standard for patients who screen positive for any DEBs. Step 4 would be necessary for patients identified as needing additional support (e.g., some patients are already receiving associated mental health care). Documentation of screening was chosen as the “measurable” outcome, as verbal screening without documentation of findings was not measurable.

The improvement team also completed a Simplified Failure Mode Effects Analysis (SFMEA) [9] to identify key drivers and potential interventions. Critical key drivers were determined to be provider competency, provider and team buy-in, appropriate screening questions, clinic flow, family comfort, family time, and provider comfort with management/referral for DEBs. Lack of a screening tool was identified as a barrier.

### 2.2 Interventions

Interventions were executed using Plan-Do Study-Act (PDSA) cycles [9].

1. Provider education: Providers met with the clinical psychologist to discuss ways to screen for disordered eating, including available pediatric screening tools that providers could choose to use. The team discussed how to document identification of disordered eating practices and when/who to refer to. In addition, nutrition and exercise goal modification was reviewed to support the patient with lifestyle changes while at the same time understanding that the primary focus of goal creation for identified patients should be healthy eating practices rather than weight loss. Providers were asked to use the information in their clinical practice going forward.
2. Implementation of paper screening tool: After initial provider education and discussion of potential screening tools, it was determined that selection of a common tool to use would be more helpful than each provider utilizing their own questions/tool. While screening for DEBs in PWM is recommended, there is no consensus to date on the ideal tool in the PWM setting [10]. For this second PDSA intervention, our team selected the Eating Disorder Diagnostic Scale-5 (EDDS-5) [11] given its ability to address limitations in existing validated screeners (e.g., SCOFF) through its comprehensive coverage of DEBs (e.g., binge eating, compensatory behaviors, and restrictive eating) and focus on asking questions associated with DSM-5 diagnostic criteria. The EDDS-5 is similar to its predecessor, the EDDS, which has been validated in adolescents [12]. We adapted the EDDS-5 further to remove stigmatizing phrases (e.g., “Have you felt fat?“) (**Se**e Fig. 1 **– EDDS-A**) and to reduce the length to make it a viable measure for a busy multidisciplinary setting. The resultant EDDS-A tool was shared with providers in paper form. A smart phrase was created within the electronic health record (EHR) to document results when using the EDDS-A and an algorithm with suggested follow-up (e.g., referral) was dispersed, based on EDDS-A results. The screener was only available in English for this QI initiative. Providers were asked to print off and use the screener with new patients at the main clinical location, where 40 % of patients in the program are seen.

**Fig. 1:**
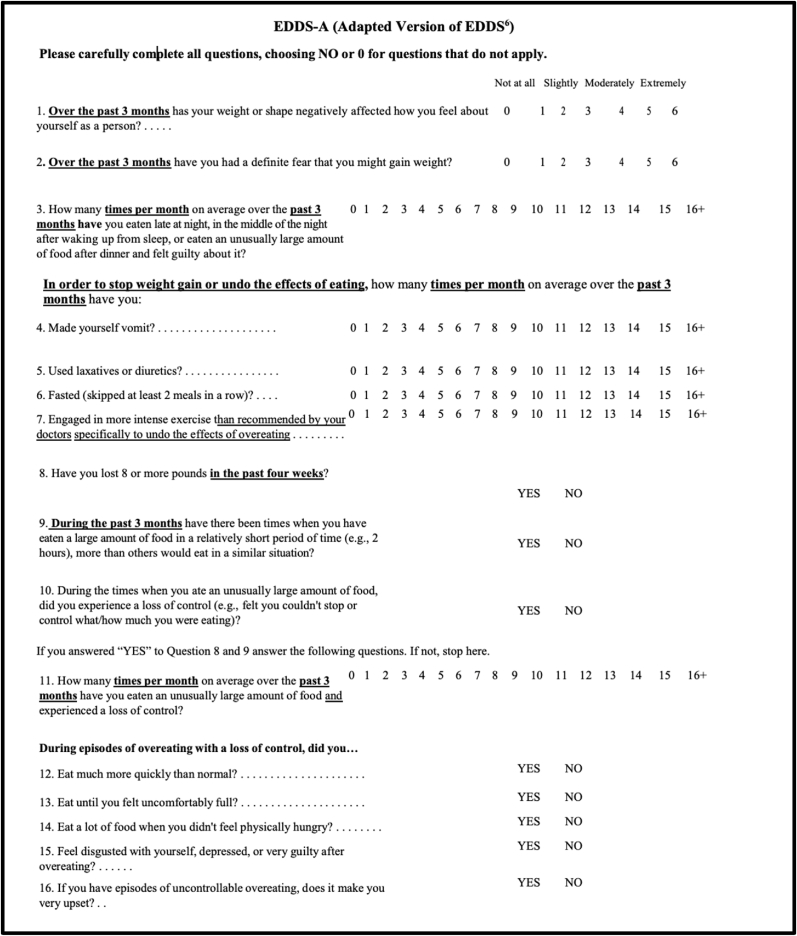
**Eating Disorder** D**iagnostic Scale** Ad**apted (EDDS-A).** Adapted from Eating Disorder Diagnostic Scale (EDDS) [11].
3. Systematic use of paper EDDS-A screener in clinics: The nurses/medical assistants on the team were asked to give the paper screener to ALL patients (≥12 years) at 3 clinic locations (accounting for ∼66 % of patients) on clinic intake, as opposed to using it at the discretion of the provider. Locations for project expansion were chosen based on staff receptivity [9].
4. Implementation of Electronic Screener: Staff buy-in and clinic flow served as barriers to consistent use of the paper screener. As such, the last PDSA involved creation of a tablet-based, electronic version of the EDDS-A that was created within the EHR in English. All PWM patients ≥12 years at all clinic locations using tablets were to automatically receive the screener as part of the pre-clinic questionnaires (i.e., 10 PWM locations; 2 PWM locations were in remote sites where tablets for intake were not available).

### 2.3 Study of the interventions

To establish a baseline, the improvement team examined medical provider notes for a six-week period (September 1 to October 12, 2022). The nine-month intervention period began October 13, 2022, and ended July 24, 2023. This QI project was reviewed by the program's Institutional Review Board and deemed exempt.

### 2.4 Measures

The primary outcome measure was the percentage of new PWM patients, ≥12 years, that had documentation of screening for DEBs in their clinical note. The number of patients with DEB screening documented (i.e., the presence/absence of at least one DEB [e.g., meal skipping to lose weight, binging, compensatory behavior]) served as the numerator. The denominator was the total number of patients meeting inclusion criteria. The primary author (RK) and a research assistant reviewed the EHR visits for all adolescents meeting inclusion criteria. Given the need for manual chart review, only new PWM patients (vs. reassessment) were included in this QI initiative.

Number of referrals to psychology and the EDP (numerator) compared to total patients meeting inclusion criteria (denominator) was a secondary measure to monitor if referrals increased in the setting of heightened DEB screening. Referral rates were dichotomized as yes (i.e. any referral to psychology or EDP) or no.

### 2.5 Analysis

Documentation of DEBs screening was plotted weekly on a Microsoft Excel annotated p-chart. Data was analyzed using standard statistical process control methods. Special cause analysis was used to identify a significant change in performance within the system, with eight or more consecutive points above or below the centerline used to cue a midline shift in the p-chart [9]. Referral data was analyzed using descriptive statistics.

## 3 Results

**Fig. 2:**
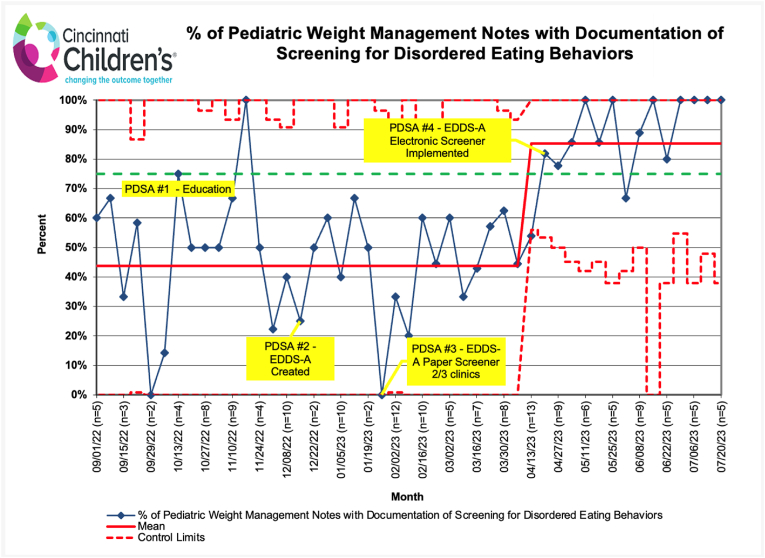
P-chart: Screening for disordered eating behaviors (DEBs) in pediatric weight management (PWM).

There were 312 patients, ages 12–18 years, seen for a new PWM visit between September 1, 2022, and July 24, 2023. The percentage of clinic notes that included documentation of screening for at least one DEB increased from 44 % to 85 % (Fig. 2). Electronic screener implementation was the PDSA that appeared to have the most impact. Analysis of provider data showed that individual provider documentation ranged from 0 to 100 % pre-PDSA cycles, with 2/3 of providers falling below 40 %. After electronic screening was implemented, 2/3 of providers were ≥80 %. Screening failures were analyzed post-hoc. Only 39 % of adolescents with Spanish-speaking guardians were given the electronic EDDS-A (in English) at check-in. The secondary measure, new referrals to psychology or the EDP, increased from 13 % at baseline to 19 % after electronic EDDS-A implementation (94 % to psychology; 6 % to EDP).

## 4 Discussion

To our knowledge, this is the first QI initiative to address provider screening for DEBs in PWM. Using QI methodology, our team increased documentation of screening for DEBs to 85 % (exceeding the goal of 80 %). EHR screening tool implementation and automaticity was the most impactful PDSA, with a level of reliability (LOR) of 2. LOR 2 interventions, which build reminders into the system making the default the desired action, lead to a more reliable process than LOR 1 interventions [13], which were utilized in the first three PDSA cycles. The overall improvement in screening is impactful from a quantitative (increased 41 %) and qualitative perspective. The 44 % of screening done at baseline was limited in scope (included absence/presence of single DEB) compared to the comprehensive screening with the EDDS-A. Additionally, there was a 46 % increase in referrals in the setting of increased screening, the majority to psychology. While causation cannot be established, the time course supports a possible correlation between heightened screening and referrals for 10.13039/100000155DEB treatment. Future research will seek to determine if enhanced screening and referrals are associated with improved patient outcomes, including decreased DEBs.

## 5 Limitations

Looking at data at weekly intervals improved feasibility given the requirement for manual chart review and allowed for rapid feedback on process issues, leading to quicker PDSA cycles. However, the weekly intervals only allowed for small sample sizes, leading to large variations in the data, especially early on. Future iterations will include screening for new and reassessment patients, which will increase sample size significantly and improve generalizability prior to spread to other programs. Additional limitations were that the EDDS-A screener was non-validated (albeit its predecessor EDDS is validated) and was only available in English. While most adolescents in our clinic are fluent in English (regardless of their guardian's primary language), post-hoc analysis revealed that adolescents with non-English speaking guardians were not consistently given the EDDS-A. Upon completion of this QI initiative, we changed the survey logic so that all adolescents received the English screener at check-in, regardless of their guardians' primary language. A follow-up review of two weeks (October 16–27, 2023) of visits showed that 95 % of new patients had DEB screening documented. Residual screening “failures” were for patients seen via telehealth or at two satellite clinics without screening tablets.

Future efforts should focus on how to equitably screen all PWM patients regardless of English fluency or appointment location/type (new vs reassessment; telehealth vs in person vs outreach clinic), given that patients from marginalized backgrounds (e.g., racial/ethnic, gender, SES) may be at higher risk for DEBs and symptoms of DEBs can vary over the course of treatment [14]. This can be done by having questionnaires available in paper form at outreach clinics and screening verbally in telehealth clinics. For patients who speak a primary language other than English, options for screening include utilizing translators to read questions to participants or having questionnaires readily available in alternate languages.

## 6 Conclusion

This QI initiative improved DEB screening in our PWM program from 44 % to 85 %. We also saw an increase in referral rates for mental health support. This study provides a road map to help PWM programs identify their own key drivers and barriers to identifying patients with DEBs who need modified care plans.

### 6.1 Key takeaways

o Consistently screening for DEBs in PWM is feasible.
o Programs should prioritize early adoption of an electronic screener for DEBs and outcomes that can be monitored electronically (negating manual review).
o Improved screening for DEBs in PWM can lead to earlier identification of high-risk patients and prompt referral to mental health practitioners.

## CRediT author contribution statement

Roohi Y. Kharofa: Conceptualization, Methodology, Validation, Formal Analysis, Investigation, Data Curation, Writing – Original Draft, Visualization. Sanita L. Ley: Conceptualization, Methodology, Resources, Writing - Review & Editing, Visualization. Kristin M. Stackpole: Conceptualization, Methodology, Investigation, Writing - Review & Editing. Jessica A. Lin: Conceptualization, Methodology, Writing - Review & Editing. Carolina M. Bejarano: Conceptualization, Methodology, Writing - Review & Editing. Katelyn Gordon: Conceptualization, Methodology, Writing - Review & Editing. Shelley Kirk: Conceptualization, Methodology, Writing - Review & Editing. Melissa Burbrink: Conceptualization, Investigation, Data Curation, Writing - Review & Editing. Robert M. Siegel: Conceptualization, Methodology, Investigation, Writing - Review & Editing.

## Disclosures

None.

## Declaration of artificial intelligence (AI) and AI-assisted technologies

The authors did not use AI during the preparation of this work.

## Financial support and sponsorship

Beyond payment to the research staff by the Cincinnati Children's Hospital Medical Center, this research did not receive any specific grant from funding agencies in the public, commercial, or not-for-profit sectors.

## Declaration of competing interest

The authors declare the following financial interests/personal relationships which may be considered as potential competing interests: Roohi Kharofa reports a relationship with Rhythm Pharmaceuticals Inc that includes: funding grants and speaking and lecture fees. If there are other authors, they declare that they have no known competing financial interests or personal relationships that could have appeared to influence the work reported in this paper.
